# Colonization of Germ-Free Piglets with Commensal *Lactobacillus amylovorus*, *Lactobacillus mucosae*, and Probiotic *E. coli* Nissle 1917 and Their Interference with *Salmonella* Typhimurium

**DOI:** 10.3390/microorganisms7080273

**Published:** 2019-08-20

**Authors:** Igor Splichal, Sharon M. Donovan, Zdislava Splichalova, Vera Neuzil Bunesova, Eva Vlkova, Vera Jenistova, Jiri Killer, Roman Svejstil, Eva Skrivanova, Alla Splichalova

**Affiliations:** 1 Laboratory of Gnotobiology, Institute of Microbiology, Czech Academy of Sciences, 549 22 Novy Hradek, Czech Republic; 2 Department of Food Science and Human Nutrition, University of Illinois, Urbana, IL 61801, USA; 3 Department of Microbiology, Nutrition and Dietetics, Faculty of Agrobiology, Food and Natural Resources, Czech University of Life Sciences, 165 00 Prague, Czech Republic; 4 Institute of Animal Physiology and Genetics, Czech Academy of Sciences, 142 20 Prague, Czech Republic

**Keywords:** *Lactobacillus amylovorus*, *Lactobacillus mucosae*, *E. coli* Nissle 1917, *Salmonella* Typhimurium, food-borne pathogen, intestine, cytokine, gnotobiotic piglet

## Abstract

Non-typhoid *Salmonellae* are worldwide spread food-borne pathogens that cause diarrhea in humans and animals. Their multi-drug resistances require alternative ways to combat this enteric pathogen. Mono-colonization of a gnotobiotic piglet gastrointestinal tract with commensal lactobacilli *Lactobacillus amylovorus* and *Lactobacillus mucosae* and with probiotic *E. coli Nissle* 1917 and their interference with *S*. Typhimurium infection was compared. The impact of bacteria and possible protection against infection with *Salmonella* were evaluated by clinical signs, bacterial translocation, intestinal histology, mRNA expression of villin, claudin-1, claudin-2, and occludin in the ileum and colon, and local intestinal and systemic levels of inflammatory cytokines IL-8, TNF-α, and IL-10. Both lactobacilli colonized the gastrointestinal tract in approximately 100× lower density compare to *E. coli* Nissle and *S*. Typhimurium. Neither *L. amylovorus* nor *L. mucosae* suppressed the inflammatory reaction caused by the 24 h infection with *S.* Typhimurium. In contrast, probiotic *E. coli* Nissle 1917 was able to suppress clinical signs, histopathological changes, the transcriptions of the proteins, and the inductions of the inflammatory cytokines. Future studies are needed to determine whether prebiotic support of the growth of lactobacilli and multistrain lactobacilli inoculum could show higher protective effects.

## 1. Introduction

A microbiota consists of a pool of microorganisms that harbor a host body. The vast majority of these microorganisms colonize the gastrointestinal tract (GIT) [1]. This resident microbiota shapes physiology of the host via digestion and assimilation of nutrients, stimulation, and maturation of host tissues, regulation of the host immune response, and keeps status quo to prevent a deleterious appearance of opportunistic and obligatory pathogens [2]. The colonization of the GIT and establishment of the balanced microbiota is sequential. In newborns, it is influenced by the mode of delivery, nutrition, and exposition to antibiotics [3]. Low concentrations of antibiotic supplements used as growth promoters in feed for livestock cause antibiotic resistance of microorganisms. Alternative supplements of the feed as acidifiers, zinc, copper, and tungsten, yeast products, nucleotides, plant extracts, prebiotics, and probiotics replace antibiotics and increase the growth of animals and feed utilization, reduce mortality and morbidity, and improve reproduction parameters without development of the antibiotic resistance [4,5].

Pioneer strains that first settled the GIT are facultative anaerobes that consume oxygen and create conditions in this environment suitable for strictly anaerobic bacteria [6]. The interactions between different members of microbial consortia and the host can extend from mutualism to pathogenesis; the interactions between different members of microbial consortia and the host can extend from mutualism to pathogenesis in dependence on the balanced microbiota composition that prevents to dysbiosis is a basic prerequisite of the host health [7].

The genus *Lactobacillus* comprises more than 200 species of Gram-positive facultative anaerobic bacteria that occupy nutrient-rich niches in humans, animals, plants, and food [8]. They are a frequent component of probiotic preparations for humans, fish, livestock, and pets [9]. *E. coli* Nissle 1917 belongs to the most used and studied probiotic bacteria. It has several siderofores and other iron acquisition systems, produce colicins and microcins, induce defensins in the host, and modulates its intestinal barrier [10,11]. In contrast to lactobacilli and probiotic *E. coli* Nissle 1917 that are considered beneficial microbes, the genus Salmonella comprises obligatory enteric pathogens [12]. Non-typhoid *Salmonellae* (NTS) are major agents of food-born infectious diarrhea and cause 200,000 deaths annually worldwide [13]. The infection with NTS commonly causes self-limiting enterocolitis (salmonellosis). However, it can cause life-threatening invasive diseases such as meningitis, osteomyelitis, septic arthritis, deep soft-tissue infection, and pneumonia in immunocompromised individuals [14,15]. The serovar Typhimurium belongs to the most commonly spread *Salmonella* serovars in human and pigs [16,17]. Due to its genetics, physiology, and anatomy, the pig is commonly used in biomedical research and it is a suitable model of human gastrointestinal [18] and infectious diseases [19]. Gnotobiotic animals, with their simple and defined microbiota, enable investigations of interactions among different bacterial species and strains and interactions between microbiota and its host [20,21,22].

The aim of this research was to evaluate the ability of the commensal *Lactobaccilus* strains, *L. amylovorus* or *L. mucosae*, to colonize the GIT of newborn germ-free piglets, their impact to the host, and their ability to suppress infection with *S*. Typhimurium. The probiotic *E. coli* Nissle 1917, which has been previously shown to reduce diarrhea in different host species, served as a positive control for protective effects against *S.* Typhimurium infection.

## 2. Materials and Methods

### 2.1. Ethics Statement

All animal experiments were reviewed and approved by the Animal Care and Use Committee of the Czech Academy of Sciences, protocol #117/2012.

### 2.2. Isolation, Characterization, and Identification of Commensal Lactobacilli

Two commensal lactobacilli were isolated from a fresh pig fecal sample using Rogosa Agar (Oxoid, Basingstoke, UK) and cultivated in enriched Wilkins–Chalgren broth (Oxoid), characterized by function tests, and identified by MALDI-TOF MS and 16S rRNA gene sequencing as described in detail in Appendix A.

### 2.3. Bacterial Strains and Bacterial Suspensions

*Lactobacillus amylovorus*, strain P1 (LA), and *Lactobacillus mucosae*, strain P5 (LB), were isolated from pig feces, characterized by function tests, identified by MALDI-TOF MS and 16S rRNA gene sequencing, and used in the experiments. A probiotic *E. coli* Nissle 1917 (EcN) is a biologically active compound of a probiotic preparation Mutaflor^®^ (Ardeypharm, Herdecke, Germany). *Salmonella enterica* subsp. enterica serovar Typhimurium, strain LT2 (*S*. Typhimurium, ST) was from a collection of the microorganisms of the Institute of Microbiology of the Czech Academy of Sciences (Novy Hradek, Czech Republic).

Fresh bacterial cultures were prepared for each experiment by cultivation for 16 h at 37 °C. Lactobacilli were cultivated in 10 mL MRS broth (Oxoid). The cells were harvested by centrifugation at 4000 × *g* for 10 min. The pellet was washed twice with 0.05 M phosphate buffer and resuspended to an approximate density of 8.5 log colony forming units (CFU)/mL. EcN and ST were cultivated overnight on meat-peptone agar slopes (blood agar base; Oxoid), and both resuspended to 8.5 log CFU/mL. The number of CFU was verified by cultivation methods.

### 2.4. Gnotobiotic Piglets

Miniature Minnesota-derived germ-free piglets were obtained by hysterectomy under inhalation isoflurane anesthesia (Isoflurane; Piramal Healthcare UK, Morpeth, UK) on the 112th day of gestation as described in details elsewhere [23]. The gnotobiotic piglets were reared in positive-pressure fiberglass isolators with heated floor. They were fed to satiety 6–7 times per day with an autoclave-sterilized cow’s milk-based formula (Mlekarna Hlinsko, Hlinsko, Czech Republic) by a nipple. Specimens taken at hysterectomy (amniotic membranes, umbilical cords, meconium, mouth, and isolator surface smears) and twice a week during rearing of the piglets (mouth, surface body and isolator smears, and stool) were cultivated for the presence of aerobic and anaerobic bacteria, and mold. Additionally, Gram-stained rectal swabs were inspected under a light microscope.

### 2.5. Experimental Design

**Figure 1:**
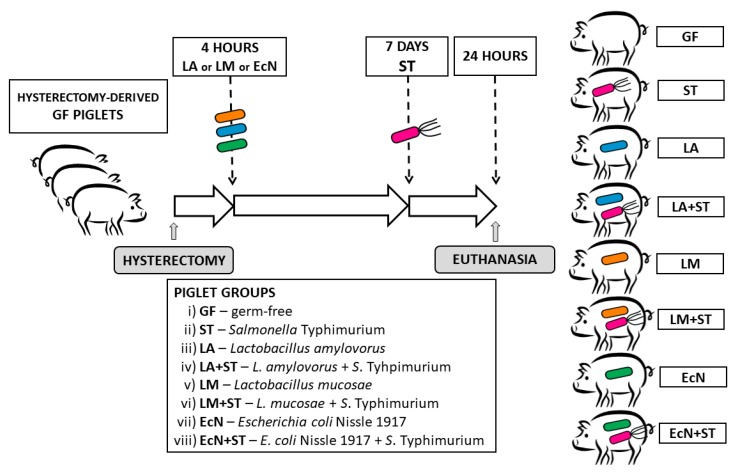
Schema of the experiment. The gnotobiotic piglets (*n* = 55) were assigned into eight groups: (i) Germ-free (GF, *n* = 6); (ii) infected with *S.* Typhimurium for 24 h (ST, *n* = 7); (iii) colonized with *L. amylovorus* (LA, *n* = 7); (iv) LA-colonized and infected with *S*. Typhimurium for 24 h (LA+ST, *n* = 7); (v) colonized with *L. mucosae* (LM, *n* = 7); (vi) LM-colonized and infected with *S*. Typhimurium for 24 h (LM+ST, *n* = 7); (vii) colonized with *E. coli* Nissle 1917 (EcN, *n* = 7); and (viii) EcN-colonized and infected with *S*. Typhimurium for 24 h (EcN+ST, *n* = 7).

Each piglet group was created from three hysterectomies (Figure 1). A total of 55 gnotobiotic piglets were assigned to eight groups: i) Germ-free for the whole experimental period (GF, *n* = 6); ii) one-week-old GF piglets orally infected with 6.0 log CFU of *S.* Typhimurium for 24 h (ST, *n* = 7); iii) orally colonized with 8.0 log CFU of *L. amylovorus* 4 h after hysterectomy (LA, *n* = 7); iv) one-week-old piglets LA-colonized (since 4 h after hysterectomy) and orally infected with 6.0 log CFU of *S*. Typhimurium for 24 h (LA+ST, *n* = 7); v) orally colonized with 8.0 log CFU of *L. mucosae* 4 h after hysterectomy (LM, *n* = 7); vi) one-week-old piglets LM-colonized (since 4 h after hysterectomy) and orally infected with 6.0 log CFU of *S*. Typhimurium for 24 h (LM+ST, *n* = 7); vii) orally colonized with 8.0 log CFU of *E. coli* Nissle 1917 4 h after hysterectomy (EcN, *n* = 7); and viii) one-week-old piglets EcN-colonized (since 4 h after hysterectomy) and orally infected with 6.0 log CFU of *S*. Typhimurium for 24 h (EcN+ST, *n* = 7). The bacterial inoculums were applied in 5 mL of a milk diet, and the control GF piglets obtained 5 mL of milk without any bacteria. Twenty-four hours after the challenge with *Salmonella* (ST, LA+ST, LM+ST, and EcN+ST), the piglets were euthanized by exsanguination via cardiac puncture under isoflurane anesthesia, and samples were collected. Their non-infected counterparts (GF, LA, LM, and EcN) were proceeded in the same way at the same age.

### 2.6. Clinical Signs

The piglets were observed for fever, anorexia, somnolence, and diarrhea during each feeding.

### 2.7. Bacterial Colonization of the GIT and Translocation

Samples of peripheral blood were cultivated log 10 and diluted by PBS. Jejunum (40 cm of the proximal part of the jejunum) and ileum (40 cm segment of a terminal part of the small intestine containing the ileum and part of the distal jejunum) lavages were cut off, filled with 2 mL of Dulbecco’s PBS (DPBS; Life Technologies, Carlsbad, CA), gently kneaded, and rinsed. Colon lavage was obtained by placing the whole colon in a 90 mm Petri dish, cut into small pieces in 4 mL of DPBS. All lavages were vigorously vortexed. Further, 0.2 g of mesenteric lymph nodes, liver, and spleen were homogenized in 0.8 mL deionized water in a 2 mL Eppendorf tube containing two 3.2 mm stainless-steel beads in a TissueLyser LT beadbeater (Qiagen, Hilden, Germany) shaken for 3 min at 50 Hz. The intestinal lavages, tissue homogenates, and blood were serially diluted in PBS and cultivated in 90 mm Petri dishes with MRS agar for lactobacilli (Oxoid), MacConkey agar (Merck, Darmstadt, Germany) for *E. coli* or Brilliant green agar (Oxoid) for *S*. Typhimurium. The plates were incubated aerobically at 37 °C for 48 h for lactobacilli or 24 h for *E. coli* or *S*. Typhimurium. The CFU were counted from dishes optimally containing 20–200 colonies.

### 2.8. Blood Plasma and Intestinal Lavage Supernatants

A citrated blood was spun at 1200 × *g* for 10 min at 8 °C, and protease inhibitor cocktail (Roche Diagnostics, Manheim, Germany) was added to the collected plasma. The intestinal lavages were spun at 2500 × *g* for 30 min at 8 °C, supernatants were filtered through 0.2 μm nitrocellulose filter (Sartorius, Goettingen, Germany), and protease inhibitor cocktail (Roche Diagnostics) was added. Both the plasma and the lavage supernatants were immediately frozen and stored at –45 °C until cytokines were measured.

### 2.9. Histologic Assessment

Terminal ileum specimens were fixed in Carnoy’s fluid for 30 min, dehydrated and embedded in paraffin. Cross-sections of tissue (5 μm) were stained with hematoxylin-eosin and examined under blinded conditions under an Olympus BX 40 microscope with an Olympus Camedia C-2000 digital camera (Olympus, Tokyo, Japan). Ten measurements for each parameter were taken per piglet to assess ileal villus length and crypt depth. Thirty evenly spaced radial lamina mucosalis propria widths per each piglet were measured. Histological scoring was evaluated as described elsewhere [21]. Briefly: i) Submucosal edema (0–2); ii) PMNs (polymorphonuclear neutrophils) infiltration into the lamina propria (0–2); iii) villus atrophy (0–3); iv) exudate in lumen (0–2); v) vessels dilatation (0–2); vi) inflammatory cellularity in lymphatic vessel lumen (0–2); vii) hyperemia (0–2); viii) hemorrhage (0–2); ix) peritonitis (0–1); or x) erosion of the epithelial layer (0–3). The total score of 0–21 points was obtained.

### 2.10. Total RNA Isolation and Reverse Transcription

Cross-sections (1–2 mm) of terminal ileum and transversal colon were stored in RNAlater (Qiagen, Hilden, Germany) at −20 °C until RNA purification. Slices of the intestine were moved from RNAlater to 600 μL RLT buffer of the RNeasy Plus Mini kit (Qiagen) containing an antifoaming reagent DX (Qiagen) and 2 mm zirconia beads (BioSpec Products, Bartlesville, OK) in 2 mL Eppendorf tube. The tissue was homogenized in TissueLyser LT beadbeater (Qiagen) at 50 Hz for 5 min at RT. The next steps of the total RNA purification followed the manufacturer’s instructions. Total RNA (500 ng) with ratio absorbances A_260_−A_320_/A_280_−_A320_ ≥ 2.0 measured in 10 mM Tris-HCl buffer pH 7.5 were reverse transcribed by QuantiTect Reverse Transcription kit (Qiagen) with initial 2 min genomic DNA wipeout at 42 °C, 20 min reverse transcription at 42 °C, and 3 min terminating step at 95 °C according to manufacturer’s instructions. Then, 180 μL of PCR quality water (Life Technologies, Carlsbad, CA) was added to 20 μL of the synthesized cDNA, and these 1/10 diluted PCR templates were stored at −25 °C until the following real-time PCR.

### 2.11. Real-Time PCR

First, 2 μL of the PCR template was added to 18 μL of the FastStart Universal Probe Master (Roche Diagnostics) containing 100 nM LNA (locked nucleic acid) probe (Universal ProbeLibrary; Roche Diagnostics) and 500 nM each of the forward and reverse primers (Generi-Biotech, Hradec Kralove, Czech Republic) (Table 1). Ten minutes initial heating at 95 °C followed 45 cycles at 95 °C for 15 s and 60 °C for 60 s. The mixtures were incubated and measured in duplicates on an iQ cycler with iQ5 Optical System Software 1.0 (Bio-Rad, Hercules, CA, USA). Cq for villin, claudin-1, claudin-2, and occludin were normalized to β-actin and cyclophilin A and their relative mRNA fold change expressions were calculated by 2^−ΔC^_T_ method [24] by GenEx 6.1 software (MultiD Analyses AB, Gothenburg, Sweden).

### 2.12. Luminex xMAP Technology

Intestinal lavage and plasma levels of IL-8, TNF-α, and IL-10 were measured by a paramagnetic sphere-based xMAP technology (Luminex Corporation, Austin, TX, USA) with a Porcine ProcartaPlex kit (Affymetrix, Santa Clara, CA, USA). The frozen samples were slowly melted at 6 °C, centrifuged at 10,000 × *g* for 5 min at 6 °C, and 25 μL of the samples were incubated with the beads according to the manufacturer’s instructions. The cytokine levels were measured on the Bio-Plex Array System and were evaluated by Bio-Plex Manager 4.01 software (Bio-Rad).

### 2.13. Statistical Analysis

Differences among the groups in parameters with normal distribution according to the Kolmogorov–Smirnov test were evaluated with one-way analysis of variance (ANOVA) with Sidak’s multiple comparisons post-hoc test or one-way ANOVA with Tukey’s multiple comparisons post-hoc test. Values that did not meet the normal distribution were evaluated with Kruskal–Wallis with Dunn’s multiple comparisons post-hoc test. The statistical comparisons were performed at *P* ˂ 0.05 by GraphPad 6 software (GraphPad Software, La Jolla, CA, USA) and differences depicted in figures by a letter system.

## 3. Results

### 3.1. Characterization and Identification of Lactobacilli

The lactobacilli suitable for the experiment were chosen according to their resistance to low pH and bile extract and strain affiliation. Strains, later identified as *L. amylovorus*, strain P1 (LA), and *L. mucosae*, strain P5 (LM), did not decrease in counts after 3 h of incubation in oxygen-free phosphate buffered saline (OF-PBS) supplemented with bile extract even at the concentration of 3% of bile extract. Strain LA was also fully resistant to pH 3, and strain LM to pH 2. LM showed a slight decrease in viability about 0.5 log CFU/mL. MALDI-TOF MS identification determined the first strain as the species *L. amylovorus* (LA) and the second one as *L. mucosae* (LM) and they were marked P1 and P5 strains for storing purposes, respectively. The identity of the selected strains was confirmed based on the 16S rRNA gene sequencing. The assigned GenBank/EMBL/DDBJ accession numbers of the 16S rRNA gene sequences are MK377165 (*L. amylovorus*, P1 strain) with 99.78% of 16S rRNA gene similarity to *L. amylovorus* DSM 20531^T^ and MK377166 (*L mucosae*, strain P5) to *L. mucosae* DSM 13345^T^ with 100%.

### 3.2. Clinical Signs

All monocolonized non-infected gnotobiotic piglets (LA, LM, and EcN) thrived and were comparable with the GF controls. The *Salmonella*-infected ST piglets suffered from fever, anorexia, somnolence, and non-bloody diarrhea. In the piglets previously colonized with LA, LM, or EcN, the infection with *Salmonella* provoked clinical signs similar to the ST group in the LA+ST and LM+ST only, but not in the EcN+ST group. The EcN+ST piglets thrived as non-infected ones.

### 3.3. Colonization of the Intestine and Translocation of L. amylovorus, L. mucosae, E. coli Nissle 1917, and Their Interference with S. Typhimurium in the Gnotobiotic Piglets

**Figure 2:**
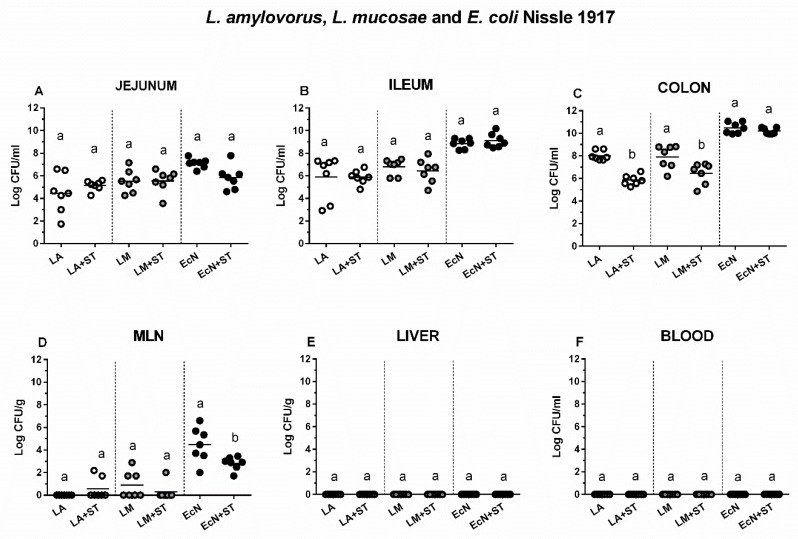
Colonization and translocation of *L. amylovorus*, *L. mucosae*, and *E. coli* Nissle 1917 in the gnotobiotic piglets. *L. amylovorus* (LA), *L. mucosae* (LM), and *E. coli* Nissle 1917 (EcN) colony forming units (CFU) were counted in the jejunum (**A**), ileum (**B**), colon (**C**), mesenteric lymph nodes (MLN; (**D**)) the liver (**E**), and blood (**F**) in monocolonized piglets (LA, LM, and EcN) and monocolonized piglets infected with *S*. Typhimurium (LA+ST, LM+ST, and EcN+ST). Interferences between LA, LM, EcN, and ST as LA vs. LA+ST, LM vs. LM+ST, and EcN vs. EcN+ST, respectively were evaluated by one-way ANOVA with Sidak’s multiple comparisons post-hoc test. Statistical differences were marked by a letter system at *P* ˂ 0.05. The same letter means no statistical significance. Log CFU are depicted as individual spots with mean as a horizontal line and *n* = 7 for all groups.

The influence of *S*. Typhimurium on the growth of *L. amylovorus*, *L. mucosae*, and *E. coli* Nissle 1917 was studied by comparison of the groups LA vs. LA+ST, LM vs. LM+ST, and EcN vs. EcN+ST (Figure 2). No differences between LA, LM, or EcN alone and LA, LM or EcN in the presence of *S*. Typhimurium were found in the jejunum (Figure 2A) and ileum (Figure 2B). In the colon (Figure 2C), the presence of *S*. Typhimurium decreased numbers of LA and LM but not EcN. Lactobacilli were not cultivated, or in some cases, seldom with relatively low numbers from MLN (Figure 2D). However, EcN translocated to MLN in all cases up to log 4 CFU/g, but *Salmonella* diminished these counts. Neither commensal lactobacilli nor probiotic *E. coli* were found in the liver (Figure 2E), spleen (not shown here), and blood (Figure 2F).

### 3.4. S. Typhimurium in the Intestine, Its Translocation, and Interference with L. amylovorus, L. mucosae, and E. coli Nissle 1917

**Figure 3:**
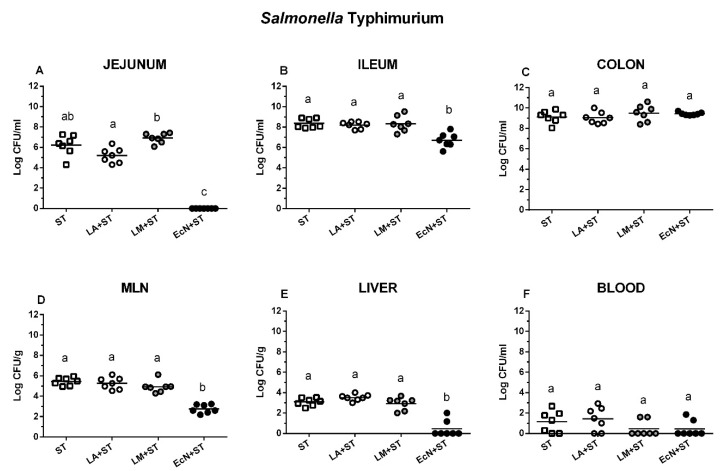
*S.* Typhimurium counts in the intestine, mesenteric lymph nodes, liver, and blood. *S*. Typhimurium (ST) colony forming units (CFU) were counted in the jejunum (**A**, proximal jejunum), the ileum (**B**), the colon (**C**), mesenteric lymph nodes (MLN; (**D**)), the liver (**E**), and blood (**F**). *S*. Typhimurium (ST) and its interferences with *L. amylovorus* (LA+ST), *L. mucosae* (LM+ST), and *Escherichia coli* Nissle 1917 (EcN+ST) were evaluated by one-way ANOVA with Tukey’s multiple comparisons post-hoc test. Statistical differences were marked by a letter system at *P* ˂ 0.05. The same letter means no statistical significance. Log CFU are depicted as individual spots with mean as a horizontal line and *n* = 7 for all groups.

*Salmonella* was completely suppressed in the jejunum in the piglets previously colonized with EcN (Figure 3A). This suppression was much lower but still statistically significant in the ileum (Figure 3B), but it did not occur in the colon (Figure 3C). Lower numbers of *Salmonella* CFU were found in MLN (Figure 3D) of the EcN+ST piglets (approximately 3 log CFU/g) but not in the cases of LA+ST or LM+ST (approximately 5 log CFU/g) that were comparable with the ST group. EcN also lowered *Salmonella* translocation into the liver (Figure 3E), but neither previous colonization with LA nor with LM did it. The lower number of bacteremia occurrence was found in LM+ST and EcN+ST groups (both in two of seven cases), but this lowering was not statistically significant (Figure 3F).

### 3.5. Histological Assessment in the Ileum of the Gnotobiotic Piglets

Histological assessment was performed on hematoxylin- and eosin-stained ileum slices. The villus height and muscular thickness were comparable among GF, LA, LM, and EcN groups (Table 2). Crypts were statistically significantly deeper and the ratio of villus height/crypt depth was lower in the EcN group compare to GF piglets.

**Figure 4:**
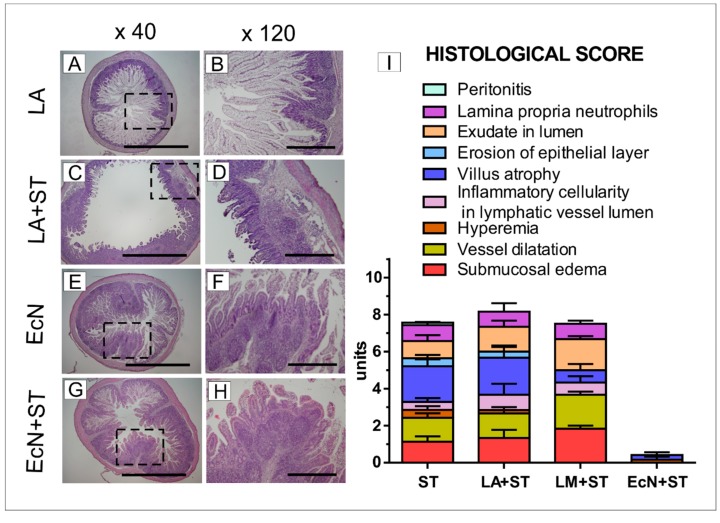
Representative hematoxylin- and eosin-stained cross sections of the ileum in the gnotobiotic piglets and a histological score. The piglet colonized with *L. amylovorus* (LA; **A**,**B**), the piglet colonized with *L. amylovorus* and infected with *S*. Typhimurium for 24 h (LA+ST; **C**,**D**), the piglet colonized with *E. coli* Nissle 1917 (EcN; **E**,**F**), and the piglet colonized with *E. coli* and infected with *S*. Typhimurium for 24 h (EcN+ST; **G**,**H**). No obvious differences were observed between the GF, LA, and LM groups; thus, LA represents these three groups. Bars represent 1 mm (**A**,**C**,**E**,**G**) and 500 μm (**B**,**D**,**F**,**H**) cross sections, respectively. Histological scores from the ileum of six piglets per group are depicted (**I**).

The ileum showed villi with the presence of vacuolated enterocytes along the whole length of the villus from the villus tips to the top of crypts in the GF, LA, and LM piglets (Figure 4A,B). The representative picture of the LA group is shown only because no obvious differences among the GF, LA, and LM groups were found. The vacuolated enterocytes were found on the apical half of villus in the EcN piglets (Figure 4E,F). *Lamina propria* cellularity in the EcN group was higher, and Peyer’s patches were 2–3-fold larger than in the GF, LA, and LM groups (Figure 4A,B). No inflammation was found in the ileum of the non-infected piglets. In contrast, the ileal sections in the piglets infected with *Salmonella* (ST, LA+ST, and LM+ST) represented by the LA+ST group (Figure 4C,D) showed signs of acute inflammation such as submucosal edema, PMN infiltration into the *lamina propria*, villus atrophy, exudate in the lumen, vessel dilatation, inflammatory cellularity in the lymphatic vessel lumen, hyperemia, hemorrhage, and multiple erosion. Using our scoring system (Figure 4I), the total histological score was between 10–12 and was comparable among infected groups except the EcN+ST group, which was without the inflammation (Figure 4G,H).

### 3.6. Transcriptions of Villin, Claudin-1, Claudin-2, and Occludin in the Intestine of the Gnotobiotic Piglets

**Figure 5:**
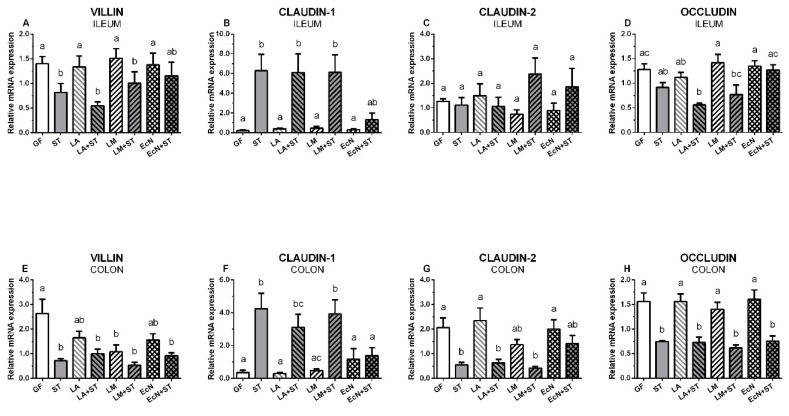
Transcriptions of villin, claudin-1, claudin-2, and occludin in the intestine of the gnotobiotic piglets. The relative mRNA expressions (fold change) were evaluated in the germ-free piglets (GF) and the piglets colonized/infected with *L. amylovorus* (LA and LA+ST), *L. mucosae* (LM and LM+ST), *E. coli* Nissle 1917 (EcN and EcN+ST), and *S*. Typhimurium (ST, LA+ST, LM+ST, and EcN+ST). Villin (**A**,**E**), claudin-1 (**B**,**F**), claudin-2 (**C**,**G**), and occludin (**D**,**H**) mRNA in the ileum (**A**–**D**) and colon (**E**–**H**) of the gnotobiotic piglets were normalized to β-actin and cyclophilin A. One-way analysis of variance (ANOVA) with Tukey’s multiple comparisons post-hoc test was used to compare differences among the groups. The values are presented as mean + SEM. Statistical differences *P* < 0.05 are denoted with different letters above the columns, and the same letter shown above the column indicates no statistically significant differences. Six samples in each group were analyzed.

Relative transcriptions of villin (5A,E), claudin-1 (5B,F), claudin-2 (5C,G), and occludin (5D,H) in the ileum (5A–D) and colon (5E–H) are depicted on Figure 5. Villin mRNA in the ileum was significantly downregulated by *Salmonella* (Figure 5A), but in the case of the piglets colonized with *E. coli* Nissle 1917 and infected with *S*. Typhimurium (EcN+ST), this decrease was not statistically significant. All bacteria downregulated villin transcription in the colon, and the presence of *L. mucosae* and *Salmonella* made this downregulation statistically significant (Figure 5E). In contrast, claudin-1 transcription (Figure 5B) was statistically significantly upregulated in the ileum in all *Salmonella*-infected piglets (ST, LA+ST, LM+ST) except the piglets colonized with EcN and later infected with ST (EcN+ST). In the colon (Figure 5F), the infection with *Salmonella* (ST, LA+ST, LM+ST) showed a similar trend as in the ileum and again with the exception of EcN of little increase transcription in comparison with GF, LA, and LM groups, but this transcription was not statistically significantly upregulated by the following infection with *Salmonella* (EcN+ST). Claudin-2 did not show any obvious trend in the ileum (Figure 5C), but in the colon (Figure 5G), *Salmonella* significantly downregulated it in the majority of *Salmonella*-infected piglets (ST, LA+ST, and LM+ST) except EcN colonized piglets (EcN+ST). In this group, the decrease was not statistically significant. As in the case of claudin-2, the down/upregulation of occludin in the ileum (Figure 5D) was not obvious and usually not statistically significant. However, in the colon (Figure 5H) the downregulation by *Salmonella* in all *Salmonella*-infected groups was observed.

### 3.7. Local and Systemic Levels of IL-8, TNF-α, and IL-10 in the Gnotobiotic Piglets

**Figure 6:**
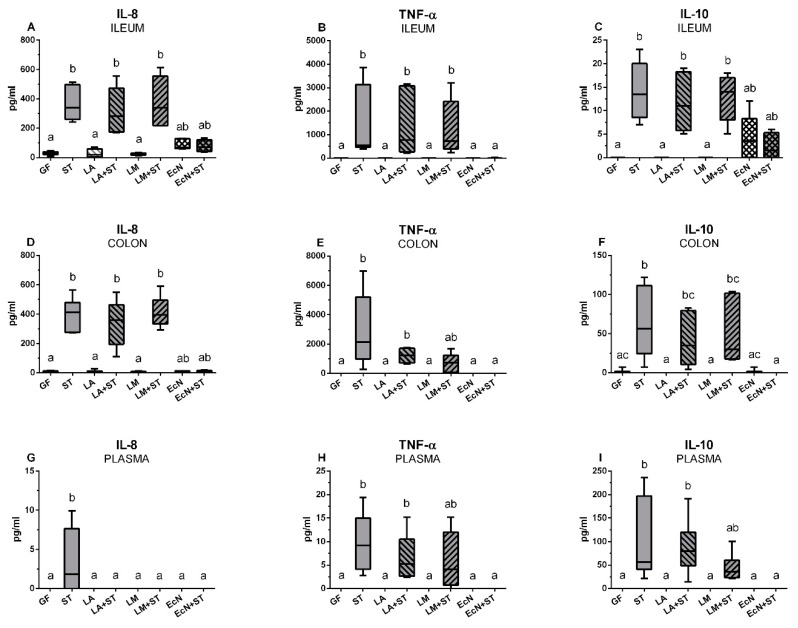
Local and systemic levels of IL-8, TNF-α, and IL-10 in the gnotobiotic piglets. The levels of IL-8 (**A**,**D**,**G**), TNF-α (**B**,**E**,**H**), and IL-10 (**C**,**F**,**I**)) in the ileum (**A**–**C**), colon (**D**–**F**), and plasma (**G**–**I**) in the germ-free piglets (GF) and the piglets colonized/infected with *L. amylovorus* (LA and LA+ST), *L. mucosae* (LM and LM+ST), *E. coli* Nissle 1917 (EcN and EcN+ST), and *S*. Typhimurium (ST, LA+ST, LM+ST, and EcN+ST) are depicted. Kruskal–Wallis test with Dunn’s multiple comparison post-hoc test was used to compare differences among the groups. The cytokine levels were measured in six piglets per group. The values are presented as median and range. Statistical differences *P* < 0.05 are denoted with different letters above the columns and the same letter shown above the column indicates no statistically significant differences.

The levels of IL-8, TNF-α, and IL-10 were measured in the ileum and colon lavages and plasma of the gnotobiotic piglets. IL-8 in the ileum and colon were comparable in the GF, LA, and LM groups and they were statistically different from the groups infected with *Salmonella* (ST, LA+ST, and LM+ST) except the EcN+ST group (Figure 6A,D). The colonization with EcN slightly increased IL-8 values in both parts of the intestine, but this increase was not significantly different from GF or from the *Salmonella*-infected piglets. A similar situation was in the cases of TNF-α levels in the ileum (Figure 5B) and colon (Figure 5E). However, TNF-α in the GF, LA, LM, and EcN groups were under the detection limit of the method. The previous colonization with EcN in the EcN+ST group was able to completely suppress TNF-α levels that were also under detection limit as in the case of the GF and other the groups mentioned above. While in the ileum the levels in the groups ST, LA+ST, and LM+ST increased significantly from the GF group (Figure 6B), in the colon, the increase in the LM+ST group was not significant (Figure 6E). The local levels of IL-10 in the intestine (Figure 6C,F) reached much lower levels than in the case of IL-8 and TNF-α, but it was again possible to observe an induced increase in the ST, LA+ST, and LM+ST groups. Additionally, in the ileum (Figure 6C), EcN induced IL-10 levels, and its previous colonization kept this level on the comparable values. However, these values were non-significant increases from the GF and other piglets infected with *Salmonella*. This increase was not observed in the colon (Figure 6F).

IL-8 in plasma was detected and significantly differed from other groups in the ST group only (Figure 6G). TNF-α was increased in ST, LA+ST, and LM+ST groups and this increase in the groups ST and LA+ST was statistically significant (Figure 6H). IL-10 showed similar results as TNF-α (Figure 6I).

## 4. Discussion

This study aimed to evaluate the growth of *L. amylovorus* or *L. mucosae* isolated from pig feces and their ability to interfere with infection by *S*. Typhimurium LT2 strain, which is virulent for gnotobiotic piglets [25]. *Lactobacillus* spp. are facultative anaerobes that are the most abundant bacteria in conventional pigs, reaching approximately 40% of the bacterial population in the small intestine [26,27]. Their percentage decreases in the colon despite increased bacterial density and species variability [26,28].

*L. amylovorus* is the most abundant species among *Lactobacillus* spp. as determined by 16S rDNA amplification and cloning, followed by *L. johnsonii*, *L. reuteri*, *L. vaginalis,* and *L. mucosae* [29,30]. It positively influenced the growth of lactobacilli in the ileum and colon of weaned piglets [30] and prevents membrane damage of an epithelial cell line infected with enterotoxigenic *E. coli* K88 [31]. *L. mucosae* is another frequent inhabitant of the swine GIT that has been tested for its possible probiotic properties [28]. It expresses a mucus-binding protein (Mub) that is typical for this species, which meditates its binding to mucus in vitro [32]. We expected that the ability of *L. mucosae* to adhere to the mucus increases the ability of this strain to colonize the intestine and create a biofilm, which limits the growth of the enteric pathogens. The commonly used probiotic *E. coli* Nissle 1917 showed a protective effect against diarrhea in infants and toddlers [33] and pigs [34]. We and others have shown that *E. coli* Nissle 1917 reduced the invasion rate of enteric pathogens in vitro [35] and alleviated signs of experimental enteric infections in the gnotobiotic piglets [20,36,37,38], including of infections with *S.* Typhimurium [25]. In the present work, *E. coli* Nissle 1917 was used for comparison of the effects of lactobacilli in the *Salmonella*-infected gnotobiotic piglets. At the same time, both *L. amylovorus* and *L. mucosae* vs. *E*. coli Nissle 1917 served for comparison of the effect of Gram-positive and Gram-negative bacteria on the host [39]. *E. coli* Nissle 1917 colonized the ileum and colon in the cell density 8 to 10 log CFU/mL. In contrast, both lactobacilli only reached 6 to 8 log CFU/mL in the ileum and colon, which appeared to be too low to effectively combat the *Salmonella* infection. While *E. coli* Nissle 1917 counts were comparable to *Salmonella* counts, *Salmonella* outnumbered lactobacilli by 100-fold. Thus, colonization with one *Lactobacillus* strain showed lower lactobacilli cell density than that found in conventional pigs, where multistrain *Lactobacillus* settlement exceeded counts of *Enterobacteriaceae* [40,41]. Infection with *Salmonella* diminished lactobacilli populations in a few gnotobiotic piglets. *E. coli* Nissle 1917 was able to suppress the growth *Salmonella* in the jejunum and ileum and suppress its translocation to the mesenteric lymph nodes and the liver; whereas neither *L. amylovorus* nor *L. mucosae* suppressed the growth of the *Salmonella* in any observed organ. In the monocolonized piglets, both lactobacilli translocated to the MLN, where they were likely trapped and eliminated. In contrast, *E. coli* Nissle 1917 showed higher ability to translocate to the MLN. However, this *E. coli* strain has semi-rough chemotype LPS that makes it complement-sensitive [42] and cannot be spread via the blood to the liver or other organs.

To investigate potential mechanisms of epithelial damage, we investigated the abundance on villin, a cytoskeletal protein that associates with the microvillar actin filaments localized in the apical border of the intestinal epithelial cells [43]. Villin also participates in the epithelial restitution after damage through its actin-severing activity [44]. Herein, transcription of villin was downregulated in *Salmonella*-infected groups with the exception of EcN+ST group, which was in accordance with the erosion of the epithelial layer in the ileum evidenced by histopathological assessment. In contrast to previous findings [37], we did not observe upregulated transcription of the villin in the intestine in the piglets colonized with *E. coli* Nissle 1917. Villin participates in the balance between actin polymerization and actin severing and facilitates the initial steps of *Salmonella* invasion [44], and *Salmonella* can spread via lymph vessels to the liver, spleen, and other organs, or it can reach these sites via blood [45].

*S.* Typhimurium causes enterocolitis (salmonellosis) in the human and pig manifested by diarrhea [16]. In this case, a movement of ions through the intestinal barrier either transporters or the lateral spaces among cells via tight junctions is altered [46]. Mammalian claudins consist of a large group of tight junction proteins. Some claudins are barrier-forming and prevent against the loss of electrolytes (e.g., claudins 1 and 4), whereas claudins 2 and 10 are pore-forming and transmit electrolytes from the intestine [47]. Therefore, we evaluated claudin-1 and claudin-2 expression with *Salmonella* to evaluate changes in the intestinal barrier. Transcription of claudin-1 was upregulated in the ileum and colon of *Salmonella*-infected piglets. Neither previous colonization with *L. amylovorus* nor *L. mucosae* prevented these increased levels of mRNA in the infected piglets.

In contrast, the previous colonization with *E. coli* Nissle 1917 prevented increased claudin-1 expression. These findings were closely correlated with diarrhea in piglets in the ST, LA+ST, and LM+ST groups, but not in the EcN+ST group. We propose that upregulated transcription of claudin-1 is an attempt to reconstitute the disrupted intestinal barrier and prevent loss of electrolytes [47]. A similar trend was found in the colon, but not in the ileum. While the small intestine is the main site of the nutrient transport, water and electrolyte transport occurs primarily in the colon [48]. The downregulated transcription of claudin-2 may be an attempt to restore the physiological function of the intestine. In contrast to claudins, much less is known about occluding [49]. While claudins participate in the transfer of low molecular compounds, occludin participate in the transport of large molecules and cells [50] and requires the proper function of the intestinal barrier. Decreased transcription of occludin in *Salmonella*-infected piglets likely reflects disrupted intestinal epithelial barrier function. This disruption was potentially to prevent ileal colonization with *E. coli* Nissle 1917, but not by the commensal lactobacilli.

Cytokines mediate communication among cell populations. They fulfill various physiological activities, but excessive secretion (‘cytokine storm’) can cause multiple organ dysfunction syndrome [51]. We focused on chemotactic cytokine (chemokine) IL-8, pro-inflammatory cytokine TNF-α, and regulatory cytokine IL-10, which are suitable diagnostic markers in enteric infections in the pig [52]. *S.* Typhimurium can facilitate its bacterial translocation either by disruption of tight junctions by direct contact with epithelium or via cytokine induction [53]. Physiologically, cytokines mainly act locally, and upregulated mRNA levels in the pig intestine infected with *S.* Typhimurium have been reported [25,54,55]. IL-8 mRNA expression in the ileum and colon of the gnotobiotic piglets were not statistically significant, however, previous colonization with *E. coli* Nissle 1917 prevented *Salmonella*-induced IL-8 mRNA in the ileum and the colon. In contrast, neither *L. amylovorus*, nor *L. mucosae* increased IL-8 mRNA expression, and their previous colonization did not prevent the increase of IL-8 mRNA in the intestine of *S*. Typhimurium-infected piglets. IL-8 induction in the ileum of the gnotobiotic piglets colonized with rough *S*. Infantis 1326/28 or *S*. Typhimurium 1591 was believed to be responsible for the protection against subsequent infection with virulent *S*. Typhimurium strains 98 [56] or LT2 [57], respectively. Intestinal expression of TNF-α in non-infected piglets or piglets previously colonized with *E. coli* Nissle 1917 (EcN+ST) was below the detection limit. While induction of TNF-α with *Salmonella* infection in the ileum was significant, expression in the colon of the piglets previously colonized with both lactobacilli was reduced, suggesting that lactobacilli partially suppressed local expression of TNF-α in the colon. We believe that increased expression of IL-8 and TNF-α in the intestine of ST, LA+ST, and LM+ST piglets exceed their physiological functions and indicate a detrimental cytokine storm [51]. The *Salmonella*-induced levels of IL-10 were much lower than IL-8 and TNF-α and probably fulfilled their regulatory functions.

IL-8 in plasma was detected in the ST group only, but values were very low. Concordantly, IL-8 levels in plasma was found in the experiment with preterm less mature gnotobiotic piglets infected with *S*. Typhimurium [21]. Lower levels of plasma TNF-α compared with our previous results [58] were found. The dose of *Salmonella* CFU used in the present experiments was two orders lower than in our former study. It may be the reason for the prolonged innate immune response and lower cytokine levels, because the maximal counts of the *Salmonella* CFU were probably reached several hours later in the piglets infected with a lower dose of *S*. Typhimurium and so these piglets were exposed to the maximal number of *Salmonella* shorter time. Increased plasma IL-10 was observed in the ST, LA+LM, and LM+ST groups. Increased sytemic levels of TNF-α and IL-10 indicated the severity of infection and poor prognosis for survival in preterm infants [59] and adults [60]. Similar prediction validity of these cytokines was reported from experiments with gnotobiotic piglets infected with necrotoxigenic *E. coli* O55 [61], in which piglets with lower clinical symptoms had significantly lower plasma TNF-α and IL-10 concentrations compared to their counterparts that suffered from a severe infection. We hypothesize that the lack of measurable systemic TNF-α and IL-10 in blood plasma in the present experiments might be due to time delayed infection, which made it possible to mobilize the innate immune response to combat to infection.

## 5. Conclusions

In the present experiments, we compared mono-colonization of the gnotobiotic piglet GIT with commensal lactobacilli, *L. amylovorus* and *L. mucosae,* and with probiotic *E. coli Nissle* 1917 and their interference with *S*. Typhimurium. Neither lactobacillus strains suppressed the inflammatory reaction caused by infection with *S.* Typhimurium, whereas, probiotic *E. coli* Nissle 1917 suppressed reduced the clinical signs and inflammatory response. Future studies are needed to determine whether higher beneficial bacteria counts could by achieved in the GIT by supporting of their growth by administering prebiotics and multistrain/species inoculum, which may more effectively inhibit enteric infections.

## Figures and Tables

**Table 1:** LNA probe-based real-Time PCR systems.

Gene	5′-forward primer-3′	5′-reverse primer-3′	#LNA Probe
BACT ^1^	TCCCTGGAGAAGAGCTACGA	AAGAGCGCCTCTGGACAC	9
CYPA ^2^	CCTGAAGCATACGGGTCCT	AAAGACCACATGTTTGCCATC	48
VILLIN	GCATGAAGAAGGTGGAGACC	ACGTTCCTCTTGCCCTTGA	42
CLD-1 ^3^	CACCACTTTGCAAGCAACC	TGGCCACAAAGATGGCTATT	3
CLD-2 ^4^	CTCGCGCCAAAGACAGAG	ATGAAGATTCCACGCAACG	77
OCLN ^5^	AAAGAGCTCTCTCGACTGGATAAA	AGCAGCAGCCATGTACTCTTC	42

^1^ β-actin, ^2^ cyclophylin A, ^3^ claudin-1, ^4^ claudin-2, ^5^ occludin.

**Table 2:** Villus height, crypt depth, ratio of villus height/crypt depth, and muscularis thickness in the terminal ileum in the gnotobiotic piglets.

	GF	LA	LM	EcN
**Villus height** (μm)	705.2 ± 76.2 ^a^	610.0 ± 289.0 ^a^	679.7 ± 82.9 ^a^	450.2 ± 146.5 ^a^
**Crypt depth** (μm)	74.1 ± 3.4 ^a^	74.5 ± 9.2 ^a^	78.8±6.6 ^a^	94.5 ± 5.0 ^b^
**Height/Depth** (ratio)	10.2 ± 2.3 ^a^	8.6 ± 5.3 ^ab^	8.7 ± 1.4 ^ab^	4.8 ± 1.6 ^b^
**Muscularis thickness** (μm)	54.2 ± 12.1 ^a^	54.2 ± 17.9 ^a^	47.6 ± 10.1 ^a^	71.2 ± 27.3 ^a^

Differences among villus height, crypt depth, ratio of villus height/crypt depth, and muscularis thickness in the terminal ileum in the germ-free (GF) piglets and the piglets mono colonized with *L. amylovorus* (LA), *L. mucosae* (LM), and *E. coli* Nissle 1917 (EcN) were evaluated by one-way analyses of variance (ANOVA) with Tukey’s multicomparison post-hoc test. The results are presented as mean ± S.D. The values with different letters significantly differ (*P* ˂ 0.05). Six piglets in each group were compared.

## Appendix A

### Methods of Isolation, Characterization, and Identification of Lactobacilli

Two different lactobacilli species were isolated from a fresh fecal sample of a one-year-old Peitrain female pig (*Sus scrofa* f. *domestic*) using Rogosa Agar (Oxoid, Basingstoke, UK) supplemented with 1.32 mL/L acetic acid (Sigma-Aldrich, St. Louis, MO, USA). Cultivation was performed in microaerophilic conditions at 37 °C for 48 h. The Wilkins–Chalgren broth (Oxoid) supplemented with 5 g/L soya peptone (Oxoid), 0.5 g/L L-cysteine (Sigma-Aldrich), and 1 mL/L Tween 80 (Sigma-Aldrich) was used for isolation of the strains and routinely sub-cultivation.

Properties predicting survival of lactobacilli during passage through the gastrointestinal tract (acid and bile tolerance) were tested as described elsewhere [62]. Briefly, overnight bacterial suspension free of cultivation media was mixed with oxygen-free phosphate buffered saline (OF-PBS) of pH 2 or 3 (pH adjusted with HCl), with OF-PBS buffer (pH 7.2) containing 1%, 2%, or 3% bile extract, or with OF-PBS buffer (pH 7.2) for control. The bacterial suspensions were incubated at 37 °C for 2 h (acid tolerance) or 3 h (bile tolerance). After the incubation, viable counts were determined by the standard plate counts methods and decrease in counts was calculated.

Both lactobacilli species were identified using MALDI-TOF MS (ethanol-formic acid extraction procedure and mixed with HCCA matrix) according to the manufacturer’s instructions (Bruker Daltonik, Bremen, Germany). Analysis of protein spectra was processed by Microflex LT MALDI-TOF MS (Bruker Daltonics) using FlexControl 3.4 software (Bruker Daltonics). Obtained spectra were analyzed using MALDI Biotyper OC version 3.1, and flexAnalysis version 3.4 (both Bruker Daltonics).

The identities of the lactobacilli strains were confirmed by 16S rRNA gene sequencing. Briefly, genomic DNA was extracted using the PrepMan^®^ Ultra Sample Preparation Reagent protocol (Thermo Fisher Scientific, Waltham, MA, USA). Fragments of the 16S rRNA gene were amplified using primers 285F (5′-GAGGGTTCGATTCTGGCTCAG-3′) and 261R (5′-AAGGAGGTGATCCAGCCGCA-3′) with PCR conditions as described elsewhere [63]. Concentrations of template DNA for PCRs ranged from 10 to 100 ng. All PCR amplifications consisted of DreamTaq Green PCR Master Mix (Thermo Fisher Scientific) and primers (Eurofins Genomics, Ebersberg, Germany) at 0.5 mol concentrations. Amplicons were purified using an E.Z.N.A Cycle Pure Kit (Omega Bio-tek, Norcross, GA, USA) and then sequenced by the company Eurofins Genomics (Eurofins Genomics). Amplicons were sequenced in both directions to achieve almost complete 16S rDNA coverage. The resulting sequences (~1450 bp) have been aligned in the Geneious version 7.1.7 software (Biomatters, Auckland, New Zealand) using Geneious Alignment. The EzBioCloud database was used to find the closest species based on pairwise similarities (%) and then deposited at GenBank database through the NCBI BankIt tool (https://www.ncbi.nlm.nih.gov/WebSub/?tool=genbank).
